# Computer Aided Design of Solvent Blends for Hybrid Cooling and Antisolvent Crystallization of Active Pharmaceutical Ingredients

**DOI:** 10.1021/acs.oprd.0c00516

**Published:** 2021-05-06

**Authors:** Oliver L. Watson, Suela Jonuzaj, John McGinty, Jan Sefcik, Amparo Galindo, George Jackson, Claire S. Adjiman

**Affiliations:** † Department of Chemical Engineering, Centre for Process Systems Engineering, Institute for Molecular Science and Engineering and EPSRC Future Manufacturing Hub in Continuous Manufacturing and Advanced Crystallisation, Imperial College London, South Kensington Campus, London SW7 2AZ, U.K.; ‡ EPSRC Future Manufacturing Hub in Continuous Manufacturing and Advanced Crystallisation, Department of Chemical and Process Engineering, University of Strathclyde, 75 Montrose Street, Glasgow G1 1XJ, U.K.

**Keywords:** crystallisation, solvent mixture, SAFT, solvent selection, solubility

## Abstract

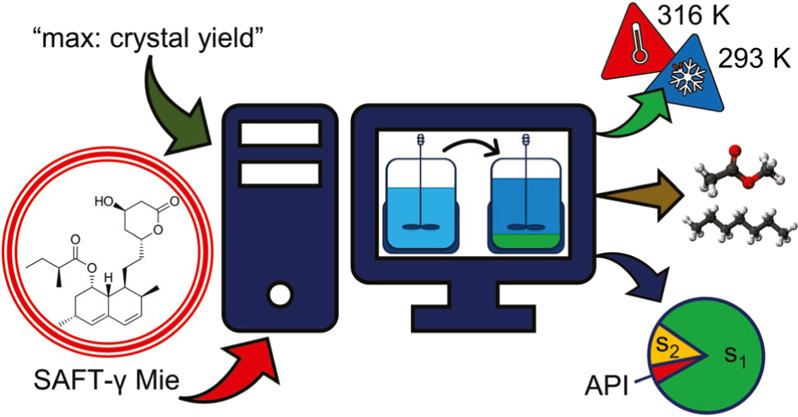

Choosing a solvent and an antisolvent for a new crystallization process is challenging due to the sheer number of possible solvent mixtures and the impact of solvent composition and crystallization temperature on process performance. To facilitate this choice, we present a general computer aided mixture/blend design (CAM^b^D) formulation for the design of optimal solvent mixtures for the crystallization of pharmaceutical products. The proposed methodology enables the simultaneous identification of the optimal process temperature, solvent, antisolvent, and composition of solvent mixture. The SAFT-γ Mie group-contribution approach is used in the design of crystallization solvents; based on an equilibrium model, both the crystal yield and solvent consumption are considered. The design formulation is implemented in gPROMS and applied to the crystallization of lovastatin and ibuprofen, where a hybrid approach combining cooling and antisolvent crystallization is compared to each method alone. For lovastatin, the use of a hybrid approach leads to an increase in crystal yield compared to antisolvent crystallization or cooling crystallization. Furthermore, it is seen that using less volatile but powerful crystallization solvents at lower temperatures can lead to better performance. When considering ibuprofen, the hybrid and antisolvent crystallization techniques provide a similar performance, but the use of solvent mixtures throughout the crystallization is critical in maximizing crystal yields and minimizing solvent consumption. We show that our more general approach to rational design of solvent blends brings significant benefits for the design of crystallization processes in pharmaceutical and chemical manufacturing.

## Introduction

More than 80% of small-molecule pharmaceuticals are delivered in a solid form,^1^ such as tablets and aerosols; because of this, pharmaceutical production is dependent on effective crystallization systems. The properties of the crystal influence not only the efficacy of the final drug product—absorption and bioavailability—but also the degree of downstream processing required due to the dependence of process performance on solid-state characteristics such as flowability and compressibility.^2^ Experience dictates that the majority of industrial crystallizers are solvent-based,^3^ in particular, due to the relative ease of operation of such units. Hence, the choice of solvent, or solvents, can drastically affect the outcome and efficiency of the crystallization process.

Thermodynamically, this impact is seen in changes to the solubility of the active pharmaceutical ingredient (API), which affects both the potential crystal yield of the API and the total volume of solvent required to perform a crystallization. These effects can be observed by changing the compound used as a solvent or when solvent mixtures are employed. Indeed, with binary mixtures, it may be possible to engineer beneficial properties, such as higher API solubility, that cannot be achieved in either of the pure solvent alone.^4^ Exploiting the enhanced performance of mixtures, however, raises many challenges for solvent selection.

The possibility of selecting a pair of solvents brings the choice of crystallization techniques to be considered into question; cooling crystallization, antisolvent crystallization, hybrid approaches, and evaporative crystallization can all be practical under the correct conditions, although the latter is not often utilized for industrial-scale processes. Which techniques are the potential solvents compatible with and how does the choice of solvent influence the feasible process conditions? The solvent mixture cannot be allowed to freeze or evaporate during crystallization, so bounds must be placed on the range of operating temperature. Similarly, the formation of two immiscible liquid phases, which may occur at certain solvent ratios, must be avoided, so the composition of solvent blends must be appropriately constrained. Beyond this, there may be health or safety concerns regarding the solvent choice^5^ or impurities within the mixture that need to be removed.

It is estimated that only 1 in every 5000 new API molecules discovered successfully completes all phases of clinical testing and progresses to market and only 1 in 25,000 recoups the initial investment.^6^ As a consequence, there are cost, material, time, and human resource constraints to contend with when developing potential pharmaceutical manufacturing processes. Nevertheless, solvent selection for crystallization systems is currently performed *via* time-consuming and expensive experiments, requiring significant materials and personnel commitments, and is heavily reliant on past experience and rules of thumb.^7^ Consequently, the full range of solvent mixtures and process conditions cannot be completely explored, and more effective crystallization systems may be overlooked.

To address these issues, a number of pharmaceutical companies have produced solvent selection guides, categorizing solvents based on health, safety, and environmental (HSE) concerns;^8−10^ an assessment of such guides has been published by the CHEM21 consortium.^11^ Whilst this approach has the benefit of being easily accessible to any scientist in a lab setting, the large volume of information presented in such documents often makes well-informed decisions difficult.

To overcome this shortcoming, Diorazio *et al.*(12) have developed a computer-based tool to account for process requirements and desired solvent properties, as well as HSE considerations, ultimately yielding a diverse shortlist of suitable solvent candidates from a much larger solvent pool. Critically, this removes some of the human decision-making inherent in previous guides, and benefits from being able to compare newer, “green” solvents to those used historically. By using experimental data, supplemented by property prediction tools, a principal component analysis (PCA) model has been proposed, providing a more interactive, graphical interface to visualize correlations between experimental properties and computed descriptors. However, whilst this approach is superior to previous solvent-selection guides, the manufacturing process is not modeled directly, instead relying on the assumption that certain combinations of physical properties will deliver the desired performance, which may not always be accurate.^13^

Over the last decades, computer-aided molecular design (CAMD) methods^14,15^ have been developed with the aim of guiding lab-based experiments toward optimal candidate molecules—in this case, crystallization solvents. These approaches are based on specific, process-derived objectives, such as maximizing crystal yield^16^ or minimizing solvent consumption,^17^ rather than on the physical properties of solvents, removing ambiguity from the design of solvent systems. Thousands of potential molecular structures are considered in CAMD problems, providing millions of possible mixture combinations and often leading to the design of novel molecules and blends. To avoid being overwhelmed by the number of possible solutions, decomposition-based solution approaches have been proposed,^18^ whereby smaller subproblems are posed and solved sequentially.

In a crystallization context, most existing methodologies have utilized a decomposition approach. First, a crystallization technique is selected, usually limiting the design to cooling crystallization in a single solvent or antisolvent crystallization operating isothermally. In both cases, the problem is centered around designing a single solvent—either the cooling crystallization solvent or the antisolvent given an initial solvent. Following this, the operating conditions are fixed, reducing the number of variables considered in the design problem, whilst also providing a means to reduce the number of solvents being considered based on their melting and boiling points. Ultimately, the objective of such an approach is to select or design a single solvent that will optimize a given criterion, such as crystal yield.^19^ Unfortunately, problem decomposition approaches may also lead to the screening out optimal solvents and solvent blends.^20^

More integrated problems, in which cooling and antisolvent effects are treated simultaneously and solvent mixtures are considered throughout,^21^ have not yet received significant attention. This is likely due to the complexity of formulating and solving a mixed-integer optimization problem to represent these design choices; such problems result in challenging nonconvex feasible regions for the continuous variables, in addition to the combinatorial growth in the solution space as more pure solvents and solvent blends are considered. Nevertheless, investigating such approaches is advantageous not only because an increased range of solvents is investigated but also because they offer the opportunity to optimize the process so that the best possible performance is derived from the solvent.

In cooling crystallization, lowering the temperature of the solvent-API mixture produces the driving force for crystallization. Intuitively, maximizing the difference between the initial and final operating temperatures should therefore maximize the reduction in the solubility of the API, thus leading to the highest possible crystal yield. However, the temperature range cannot be made arbitrarily large, due to the freezing and boiling points of the solvents present—their liquid range. As such, whilst fixing the initial and final temperature of the system removes two degrees of freedom from the problem, the choice of fixing the temperature range will also screen out any solvent from the design problem that has a liquid range outside of the set temperature range. Because higher process temperatures eliminate the opportunity to select more volatile, but potentially powerful, solvents, whilst also leading to higher energy costs, it may not always be optimal to begin the crystallization at the highest allowable operating temperature; optimal solutions may be overlooked when fixing the crystallization temperature during solvent selection.

Similarly, the approach taken in the antisolvent design workflow may obstruct the useful application of solvent blends and the nonlinear solubility behavior that they can promote. One such example is the solubility of paracetamol. It is well understood that paracetamol is only sparingly soluble in water; despite this, the addition of water to pure acetone initially increases the solubility of paracetamol in the mixture.^4^ Indeed, whilst the solubility of paracetamol in pure acetone and pure water is 94.5 and 14.0 g/kg, respectively, it is possible to achieve a solubility that is at least 4.5 times larger than in pure acetone by considering a 70:30 mixture by mass of acetone and water (at 296.15 K). If one were to attempt an antisolvent crystallization process by starting with pure acetone, as is typical in a decomposition-based approach, a much greater volume would be required to generate supersaturation; at low water volumes, the paracetamol would simply be diluted by the addition of water, resulting in lost capacity of the crystallizer. However, by considering all possible compositions of acetone and water to dissolve the required quantity of paracetamol and thus starting from an elevated solubility of paracetamol, water can then be used as a powerful antisolvent to achieve both a high crystal yield and a lower solvent consumption.

Furthermore, a hybrid crystallization technique that integrates cooling and antisolvent crystallization can provide additional benefits. Operating at a higher initial temperature makes use of the higher API solubility, reducing solvent consumption and increasing crystal yield following the subsequent reduction in temperature and addition of antisolvents. As such, the design of integrated crystallization methods, where cooling and antisolvent techniques can be applied simultaneously, is important for improving the efficiency of the crystallization process. This has been investigated in more general computer-aided mixture/blend design (CAM^b^D) formulations, where a “generate-and-test” methodology has been applied^22^ to solve the problem, screening all possible solvent combinations. Whilst this improves the likelihood of finding a globally optimal solution to the solvent design problem, the computational time increases rapidly with the number of solvent candidates considered; 10 potential solvents result in only 45 binary solvent mixtures, but a list of 100 solvents gives 4950 possible combinations. Physical insights could be used to reduce the number of options, for example, by only pairing solvents from different families—such as aliphatics, alcohols, and acetates—but the design space would nevertheless remain intractably large. As such, “generate-and-test” approaches have limited flexibility for larger numbers of solvents; trailling a number of different design objectives, or different process constraints, may not be feasible.

Recently, the use of generalized disjunctive programming (GDP)^23^ within the CAM^b^D framework—used to formulate logical constraints as mathematical expressions in the optimization problem—has been proposed,^24^ showing that solvent blends can provide optimal conditions to maximize the solubility of active pharmaceutical ingredients.^25^ This concept has been further developed by Watson *et al.*,^21^ where integrated techniques for crystallization have been explored and optimized. The promising results obtained warrant further development of comprehensive CAM^b^D formulations.

In our current paper, a CAM^b^D formulation for the design of integrated crystallization solvent systems (without evaporation) is proposed, whereby the identities of the solvent and antisolvent molecules are optimized, alongside their compositions and the process operating temperatures. This overcomes the potential limitations in the current decomposition-based approaches. The design formulation is implemented in gPROMS and applied to the crystallization of lovastatin and of ibuprofen, whereby the effects of crystallization technique, process temperature, and solvent specification are explored.

## Methodology

**Figure 1:**
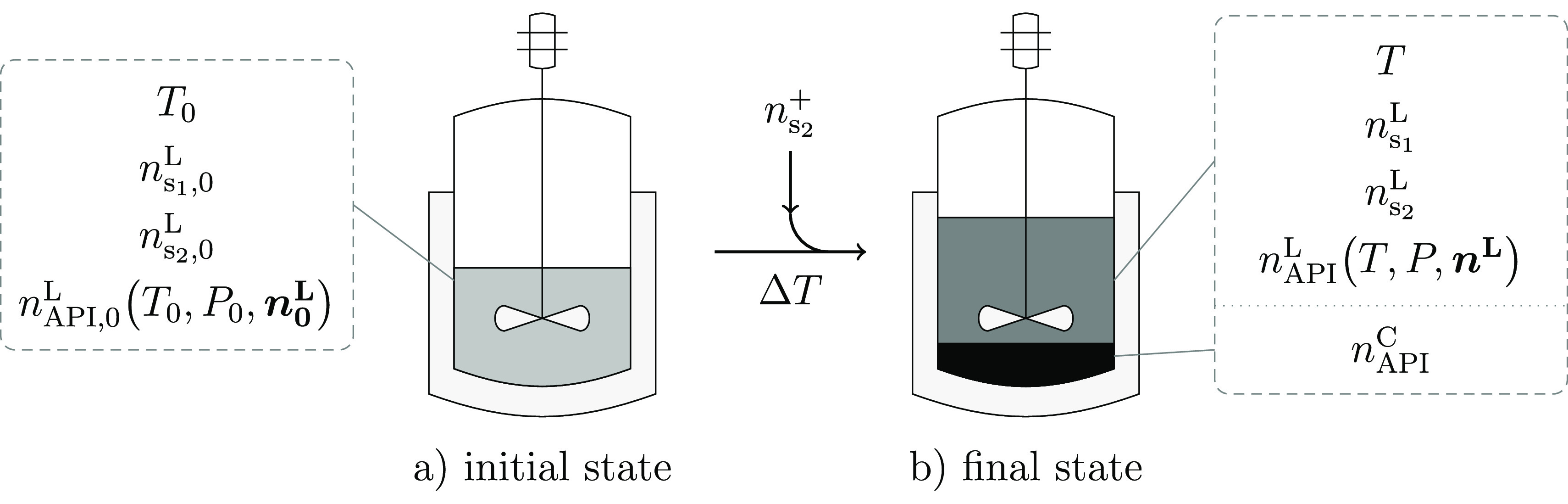
The crystallization process, as described in the general formulation. From left to right, the process transitions from (a) the initial state (subscript 0), where the solvent blend is saturated with the API solute but no crystals are present, to (b) the final state, generating a solid phase of API by reducing the operating temperature by Δ*T* = *T*_0_ – *T*, by introducing additional moles of antisolvent , or by doing both. In (b), the final solvent mixture is also saturated with the API solute, which is in a state of solid–liquid equilibrium. Here, *n*_i_^ϕ^ denotes the moles of component i in phase ϕ ∈{L, C}, where L and C refer to the liquid and crystal phases, respectively. The API is assumed to be the only component in solid–liquid equilibrium, which is a function of temperature *T*, pressure *P*, and liquid composition ***n***^L^. Impurities in the mixture are assumed to be negligible.

The CAM^b^D problem is based on a generic formulation for the design of integrated crystallization solvent systems, whereby optimal solvent and antisolvent molecules (s_1_ and s_2_, respectively), their compositions, and the process temperatures are identified. The approach to design focusses on the thermodynamic, rather than kinetic, aspects of crystallization, and hence, only information about the initial and final states of the system is required, as illustrated in Figure 1. In the initial state (Figure 1a), all of the API, *n*_API,0_^L^ (mol), is dissolved in the initial solvent blend, comprising of  moles of s_1_ and  moles of s_2_, at a temperature *T*_0_ (K), producing a saturated solution. The process conditions are adjusted to generate the driving force for crystallization and reach the final state of the system (Figure 1b)), assumed to be the point when solid–liquid equilibrium (SLE) is reached between the *n*_API_^C^ moles of API crystals and the remaining API dissolved in the final solvent mixture, consisting of *n*_API_^L^ moles of API,  moles of s_1_, and  moles of s_2_, at a final temperature *T*. It is assumed that there is no solvent loss so  and .

From this description, two key design objectives—API crystal yield and solvent consumption—can be considered, without knowledge of the specific path taken between the initial and final state. In the proposed formulation, the use of solvent blends in both the initial and final states of the system is permitted, provided that the blends consist of the same two solvent molecules, the “solvent” s_1_ and the “antisolvent” s_2_, in different proportions. It is then possible to exploit an enhanced solubility in the initial blend,^4^ reducing the volume of solvent required to dissolve the required quantity of API, followed by an extreme reduction in API solubility through the addition of more antisolvent, thus achieving larger crystal yields than when starting with a pure solvent. Additionally, concurrent cooling and antisolvent crystallization are permitted within the design, utilizing the benefits of both crystallization techniques in the optimization, as well as the potential synergistic interactions between the two modes of operation.

**Figure 2:**
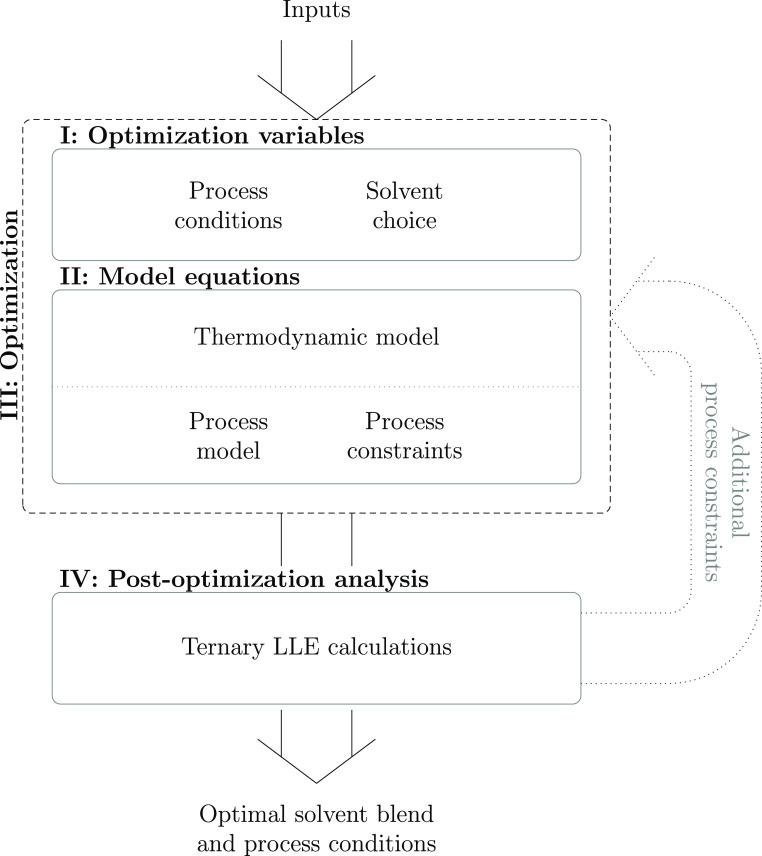
Overview of the structure of the solvent design framework. In “Block I: Optimization variables”, two sets of optimization variables are defined: the process conditions (operating temperatures and solvent composition) and the choice of solvent and antisolvent. In “Block II: Model equations”, the general model is described by five sets of equations: the objective function; the thermodynamic model consists of SAFT-γ Mie, to calculate the thermodynamic properties of the liquid phases, and the SLE model to predict the solubility of the API; solvent assignment constraints; the process model; and design constraints that exclude impractical solutions or set design targets. This information is combined in “Block III: Optimization” to obtain the MINLP to be solved, outputting the optimal process temperature, solvent blend, and blend composition for the initial and final state of the crystallization. Additional calculations are performed in “Block IV: Post-optimization analysis” to determine whether the resulting ternary mixture exhibits liquid–liquid equilibria (LLE). If so, additional process constraints are imposed for that specific solvent blend and the optimization is re-run.

To be readily applicable to the pharmaceutical industry and to facilitate the rapid deployment of novel drug molecules, the proposed methodology is based on a “plug-and-play” approach; users can input their chosen API, specify lists of potential solvent candidates, and adjust the objectives of the optimization problem quickly and easily. The optimization problem can be split into the three main sections depicted in Figure 2—model equations, optimization variables, and the optimization itself—to generate optimal solvent blends and process conditions to suit the user’s needs.

### Block I: Optimization Variables

The first group of optimization variables is a set of discrete variables that represent the selection of the solvent and antisolvent molecules (s_1_ and s_2_ respectively) that constitute the solvent blend. These are chosen from a list of possible solvent candidates supplied by the user, denoted by the set *N*_S_, and are represented mathematically by the binary (0, 1) variables *y*_ii,*j*_, where ii is either s_1_ or s_2_, and *j* is selected from *N*_S_. During the optimization, each combination of candidate solvents s_1_ and s_2_ can be switched on or off. For example, if acetone were selected as the solvent, and water as the antisolvent, this would correspond to *y*_s_1_,acetone_ = 1 and *y*_s_2_,water_ = 1, with all other combinations being switched off (*i.e.*, the relevant binary variables are set to zero).

The remaining variables represent the process conditions within the crystallizer—the initial and final temperatures of the crystallization process, *T*_0_ and *T*, respectively, along with the initial and final compositions of the binary solvent mixture,  and , where the superscript S refers to the fact that only the solvent/antisolvent is included in this binary mixture, and the final number of moles of antisolvent present, . By defining the number of moles of antisolvent, in conjunction with the final composition of the binary solvent mixture, this also defines the total number of moles of solvent in the system.

It should be noted that, within the general formulation, there is no specific variable to represent the selection of a crystallization technique. Instead, the optimal technique, or combination of techniques, is an inherent outcome of the optimization problem. For instance, if a higher crystal yield can be obtained from simply cooling a solvent–API mixture, compared to cooling and adding antisolvent to the mixture, then the optimization solver will return cooling crystallization, and not hybrid crystallization, as a solution: the number of added moles of antisolvent added, denoted here as , will be zero and the initial and final temperatures will be different. Thus, there is an *opportunity* to generate a solution that represents cooling crystallization, or antisolvent crystallization, or a hybrid of the two techniques, without the need to specify this *a priori*. Equivalently, in situations where the use of a pure solvent outperforms a solvent mixture, the mole fraction  of the second solvent is found to be zero throughout the crystallization. If required, the user can introduce additional constraints to limit the crystallization to a single technique; these are discussed in the case studies.

The composition of the binary solvent mixture is simply bounded by the definition of mole fraction
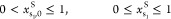
1with other constraints ensuring that solvent s_1_ is always present.

The bounds placed upon the process temperature are less obviously defined. In practice, there will always be upper and lower limits to the operating temperature, *T*_max_ and *T*_min_:
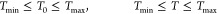
2The values of these bounds are set based on the practicality and feasibility of the given crystallization process. For example, certain pharmaceutical molecules may degrade at high temperatures, the safety procedures in place for a crystallization vessel may not be sufficient for temperatures above a specific threshold, or it may be too costly to cool the solvent-API mixture below the temperature of standard cooling water.

Whilst there is a clear physical lower bound to the final number of moles of antisolvent in the solvent mixture, there cannot be a negative number of moles
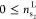
3it is likely that this value will also be bounded by the design constraints, such as the maximum or minimum limit of solvent consumption or the condition preventing evaporative crystallization (eq 20).

### Block II: Model Equations

#### Objective Function

“Block II: Model equations” in Figure 2 defines the set of equations that are required to calculate the objective function—in our work, the focus is on maximizing the crystal yield of the API, *Y*_API_
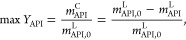
4where *m*_i_^ϕ^ refers to the mass of component i in phase ϕ, either the liquid phase (L) or the crystal phase (C), and subscript “0” refers to the initial state throughout.

The choice of objective of the optimization problem is flexible, however, and eq 4 can be interchanged with other functions, such as the minimization of solvent consumption χ_s_ (g of solvent per g of API), or *V*_s_ (mL of solvent per g of API):
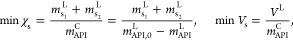
5where the total liquid volume is denoted by *V*^L^ (in L) and is calculated through the thermodynamic model.

#### Thermodynamic Model

So that the objective function can be computed, the relationship between the properties of interest and the temperature, pressure, and composition of the liquid mixture and the API must be determined. Because the API in the liquid phase is assumed to be at equilibrium with the crystal phase, the thermodynamic properties of the solvent-API mixture are required in order to predict the API solubility. In our work, the statistical associating fluid theory (SAFT) is selected. Specifically, the group-contribution (GC) version of the equation of state (EoS) based on the Mie potential, SAFT-γ Mie,^26^ is employed. For the SAFT-γ Mie group parameters used throughout this work, refer to Tables 8−10 in the Appendix.

The use of GC methods within the thermodynamic model supports a “plug-and-play” approach, allowing the user to describe the relevant API and solvent molecules in terms of functional-group “building blocks”. The thermodynamic model is not necessarily limited to molecules for which experimental data are readily available. Hutacharoen *et al.*,^27^ Di Lecce *et al.*,^28^ Febra *et al.*,^29^ and Haslam *et al.*(30) have recently shown that, thanks to the rigorous thermodynamic concepts that the SAFT-γ Mie EoS, the thermodynamic platform can provide high-quality predictions of the solubility of pharmaceutical compounds in a range of solvents, as well as liquid–liquid and vapor–liquid equilibria, all key properties for industrial applications.

As the formulation of the crystallization process is based on an equilibrium model—both the initial and final states of the system denote solvent blends saturated with API solute (Figure 1)—it is necessary to be able to predict the solubility of the API under all possible process conditions. It is assumed that only the API undergoes a phase change. The process operating temperature is prevented from approaching the melting temperatures of the solvent and antisolvent *via* constraints specified later in this section, and it is assumed that no solvates.

Thus, the solubility of the API in a solvent blend is determined from
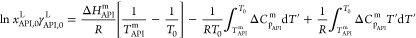
6
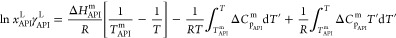
7where γ_API,0_^L^ (γ_API_^L^) is the liquid-phase activity coefficient of the API in the initial (final) liquid phase, calculated with SAFT-γ Mie as a function of *T*_0_ (*T*), the initial (final) process operating temperature, *P*_0_ (*P*), the initial (final) pressure, and *x*_i,0_^L^ (*x*_i_^L^), i ∈ {API, s_1_, s_2_}, the initial (final) liquid-phase mole fractions; *T*_API_^m^ is the melting temperature and Δ*H*_API_^m^ the enthalpy of melting of the API, which are assumed to be known beforehand, preferably from experiments;  is the difference in the isobaric specific heat capacity of the API between the solid and liquid phase.

The entropic contribution in the SLE equations, given by the last term in eqs 6 and 7 and expressed in terms of the heat capacity difference, is often ignored^16^ under the assumption that the two integrals in eq 6 or in eq 7 are approximately equal or that the heat capacity difference is close to zero. However, this contribution has been shown to have a significant impact in certain cases,^31−33^ which cannot necessarily be identified *a priori*, so we include it here. It can be omitted when the required heat capacity data are not available.

#### Solvent Assignment Constraints

A number of logical constraints are imposed on the binary variables representing the choice of solvent, namely:

- both solvent and antisolvent should each consist of one solvent candidate only

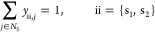
8

- no solvent candidate should be used as both solvent and antisolvent

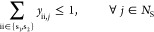
9

Furthermore, it is necessary for the binary variables *y*_ii,*j*_ to be combined with a description of the candidate molecule, j, in question. To this end, each solvent candidate is defined as a combination of functional groups, selected from a database, expressed by the parameter ν_*j*,k_, where k is a functional group in set *N*_FG_. For instance, heptane would comprise two CH_3_ groups and five CH_2_ groups (ν_heptane,CH_3__ = 2 and ν_heptane,CH_2__ = 5, respectively). The solvents s_1_ and s_2_ are then defined in terms of these functional groups as follows
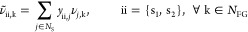
10where ν̃_ii,k_ is the number of functional group k present in solvent ii.

#### Process Model

For a successful crystallization, it is clear that the mass of the API solute in the final state should be lower than that in the initial state, in order to transfer solute into the crystal phase. As such, mass balances are performed on the initial and final states of the system, subject to the thermodynamic equilibrium between the solid and liquid phases (eqs 6 and 7). First, the mole fractions are related to mole numbers as follows
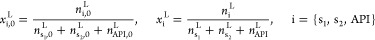
11

Next, the number of moles of solvents s_1_ and s_2_ in the initial and final states of the system are connected:
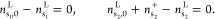
12

The moles *n*_API_^C^ of crystal produced can be calculated by taking the difference between the number of moles of the API solute in the initial state and in the final state.
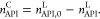
13A simple conversion factor can be used to determine the crystal mass, *m*_API_^C^, by using the molecular weight of the API in question, *M*_API_
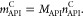
14

#### Design Constraints

Having selected an objective function, one can then specify additional constraints on the process and solvents, beyond those already defined within the process model. For instance, in an optimization problem aiming to maximize crystal yield of the API, one may decide to limit the solvent consumption to no more than a specified level. Alternatively, if the intention of the optimization is to design a more environmentally sustainable crystallization process, one could minimize solvent consumption, but ensure that the crystal yield is greater than a minimum level, for example, 90%. Further heuristic constraints can be included for this purpose too; for instance, experience may dictate that operating below 4 mL solvent/(g crystal) is likely to lead to an inoperable process due to high viscosity and shear forces, so the specific volume of solvent can be bounded by this value, preventing the optimization from reducing the total volume of solvent beyond feasible physical limits.

Whilst certain solvent blends may produce mathematically optimal results with respect to API crystal yield or solvent consumption, it is also important to understand whether a solvent pairing is feasible under the selected process conditions. Three key conditions must be satisfied when choosing solvents for the crystallization problem: a solvent should not boil or freeze during the crystallization process and, in the case of solvent mixtures, the solvents should also be miscible with each other over the process conditions.

Liquid-range constraints are enforced for each solvent candidate depending on two optimization variables, the initial and final operating temperatures, based on the (experimental) melting point of each solvent *j*, *T*_*j*_^m^, where all temperatures are assumed to be in K,
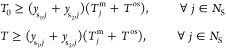
15and the bubble point of the solvent mixture, *T*_mix_^b^
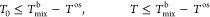
16where *T*^os^ is a temperature offset chosen to prevent the process from operating too close to a solvent phase change.

One could widen the feasible temperature range by calculating the melting point of the solvent mixture, thereby making it possible to take advantage of eutectic behavior. This formulation is however not considered here as a relatively high value of 293.15 K is used as a lower bound on the crystallization temperature.

Using solvent-dependent bounds for the operating temperatures, rather than fixed maximum and minimum temperature bounds as in previous work,^16^ avoids the screening out of potentially effective yet relatively volatile solvents. Additionally, while previous work in this field has been reliant on the boiling points of pure solvents to define the upper limit of the liquid range of the mixture, the bubble point of the liquid mixture is calculated using the SAFT-γ Mie EoS in our formulation. This ensures that any nonlinearities, such as the formation of high- or low-boiling azeotropes, are captured in the optimization. This may allow higher temperatures to be utilized during operation or conversely limit the highest allowable temperatures of mixtures of solvents relative to those possible with pure solvents. Finally, the formulation also includes a buffer (taken as *T*^os^ = 10 K in our current work) to avoid cases where, during operation, disturbances to the process temperature may otherwise push the system beyond the bubble point or freezing point of a solvent mixture.

To ensure the solvent mixture forms a single, stable liquid phase, the miscibility of the solvent blend is assessed by using of the Gibbs stability criterion^34^ for which an explicit (algebraic) expression exists in the case of binary mixtures. The stability of the binary solvent–antisolvent mixture (denoted by adding a superscript S to the mole fractions) is calculated by examining the derivative of the chemical potential of solvent s_2_ in the binary solvent mixture, :
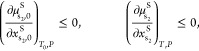
17where the mole fractions in the binary mixture are defined by the following set of equations
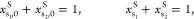
18
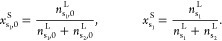
19

Whilst these constraints provide the necessary and sufficient conditions to prevent the instability of the solvent pair in both the initial and final states of the system, it does not necessarily guarantee the miscibility of the ternary mixture that includes the API. Including a constraint to ensure the miscibility of the ternary mixtures is more challenging as there is no explicit criterion in this case. However, in most cases, the API does not affect the calculated phase stability significantly, as it is often present in low concentrations relative to the solvent and antisolvent; its presence is therefore neglected in the optimization. This assumption is then validated in “Block IV: Post-optimization analysis”, after a solution to the crystallization design problem has been found.

A final constraint must be included; as the formulation does not allow for evaporative crystallization, the antisolvent cannot be removed from the system. This is accounted for in eq 20:
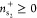
20

### Block III: Optimization Solution

The optimization formulation is solved using an algorithm such as Outer-Approximation^35^ or Branch & Bound.^36,37^ It is instructive to generate multiple high-performance solutions, obtained using integer cuts, and to undertake parametric studies, for example, based on varying the bounds on the operating temperature.

### Block IV: PostOptimization Analysis

Once the optimization (Block III) is complete, a simple analysis of the solutions should be performed as a final feasibility check to determine whether the ternary mixture is stable or whether it will separate into two distinct liquid phases, with the potential for the formation of a solid phase too. The existence of such LLE for a given ternary mixture composition is tested within gPROMS.

Upon performing this calculation, two scenarios can arise. First, only one liquid phase is found to exist for the ternary mixture under the optimal process conditions found during the optimization solution, validating the assumption that the liquid phase is stable even when the API is present—in this case, the solution can be taken to be trailled in an experimental setting. However, in the second scenario, where the suggested ternary mixture comprises two distinct liquid phases, the assumption that the solvent mixture stability is a good indicator of overall mixture stability is incorrect and should be revisited.

There are a number of approaches available for adjusting the optimization problem to account for the existence of LLE in the final solution. Here, we consider two simple methods that can be quickly implemented into the generic optimization problem. One option is to remove the solvent pair that formed the LLE (denoted solvent A and antisolvent B here) from the optimization problem entirely:
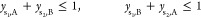
21The use of the binary variables *y*_ii,*j*_ in this way allows both candidate solvents, A and B, to be selected with other solvent candidates but not together.

Alternatively, one can introduce constraints on the binary solvent composition to avoid the LLE region. In this case, the LLE envelope first needs to be determined. Because the initial and final states of the crystallization mixtures are considered to be at SLE, only two stability limits on the envelope are important for a fixed temperature and pressure, at the initial and/or final state. This can be understood by applying the Gibbs phase rule, *F* = *C* – *P* + 2, where the degrees of freedom *F* of a system can be determined from the number of components, *C*, and phases, *P*, present. In the case of the crystallization process considered in this formulation, there are three components (s_1_, s_2_, and API) and two phases (liquid and solid), giving three degrees of freedom (temperature, pressure, and the ratio of the mole fractions). When a second liquid phase is included, the number of degrees of freedom is reduced to two, meaning that the compositions of both liquid phases at solid–liquid–liquid-equilibrium (SLLE) are fixed by setting the temperature and pressure.

As such, the composition of the two liquid phases, denoted here as α and β, can be found by determining the two points on the LLE envelope that intersect the API solubility curve. These limits can then be used to further constrain the optimization problem to avoid the LLE region:
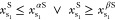
22
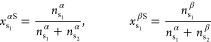
23where α is taken to be the antisolvent-rich phase, whilst β is the solvent-rich phase, and as such, these constraints hold when . Because feasible operating points may exist anywhere outside the SLLE envelope, two revised optimization formulations should be solved—one in the antisolvent rich region , and one in the solvent rich region  It should be emphasized that the SLLE limits obtained in this manner are only valid for the chosen temperature, and any changes to the optimal temperature in subsequent optimizations would require this process to be repeated.

## Results and Discussion

The formulation is implemented in gPROMS version 6.0.2, applying gSAFT to perform calculations using the SAFT-γ Mie group-contribution thermodynamic platform. Two case studies are considered, pertaining to the crystallization of lovastatin and ibuprofen. The effects of the crystallization technique, the operating temperature range, and the mass of solvent consumed on the optimal solvent choice, compositions, and process temperatures are all considered in an integrated manner. In all cases, the operating pressure is taken to be 1 atm. In order, to explore fully the range of solvents that maximize crystal yield, no upper limit is placed on solvent consumption, but this can be easily implemented to reduce the design space and eliminate potentially impractical solvent mixtures.

### Case Study I: Crystallization of Lovastatin

**Figure 3:**
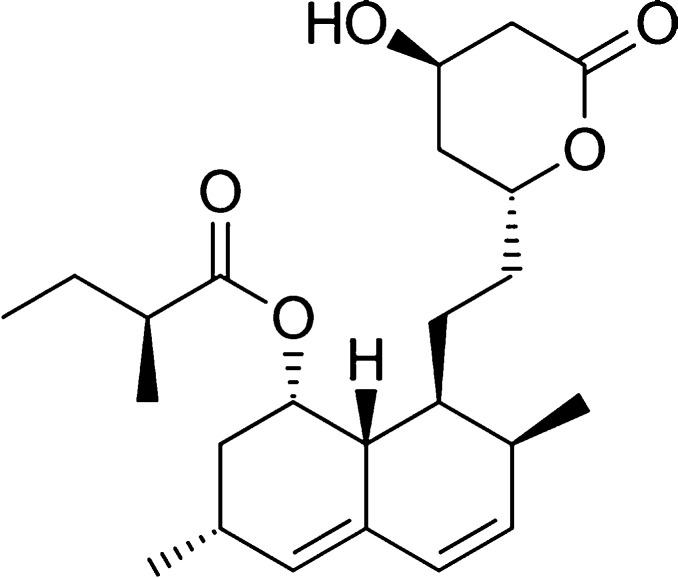
Chemical structure of lovastatin, the API considered in Case Study I.

Lovastatin, as with all statin medications, is used to lower the levels of low-density lipoprotein cholesterol in the blood, thus reducing the risk of cardiovascular disease. Comprising a hydroxylated heterocyclic lactone group, an unsaturated bicyclic structure and a branched butanoate ester (Figure 3), the complex interactions of the API with solvents make it difficult to predict the phase behavior of the mixture; following the oft-touted “like-dissolves-like” heuristic may not yield practical solvent mixtures for crystallization. The SAFT-γ Mie group-contribution approach has been shown to yield accurate predictions of the solubility of lovastatin in linear alcohols and ethyl acetates across a range of temperatures.^38^ For the model used here (shown in the Appendix), we find a root-mean-square error in the logarithm (log_10_) of 0.30 for the solubility (mole fraction) of lovastatin in 15 solvents, when comparing experimental data to the predicted values. Such an accuracy is typically sufficient for the ranking of solvents. The performance of the approach is particularly accurate for larger solvents (3 or more carbons), as can be expected from a group-contribution method.

Perhaps the most important use of a computer-aided approach is the capacity to identify quickly several high-performance solutions to the solvent design problem, before trialling them experimentally. In practice, this should reduce the amount of time and material spent testing ineffectual crystallization solvent blends. Therefore, the generic formulation is applied here to rank solvent mixtures, based on maximizing the crystal yield of lovastatin that can be achieved. For all solutions, the post-optimization analysis (see Figure 2) confirms that there is only one liquid phase in the initial and final states of the system.

#### Comparison of Traditional Approaches and Hybrid Crystallization

Whilst it is trivial to state that using a hybrid approach (H) to crystallization can achieve better performance than cooling or antisolvent crystallization alone, it is important to quantify the degree of improvement that is possible. The use of solvent mixtures complicates solvent recycling processes; the blends must first be separated and purified before being reused. Similarly, combining cooling and antisolvent crystallization increases the complexity of the crystallization process, placing greater emphasis on bespoke crystallizer design and control systems, along with the requirement for a higher level of operator expertise. Ultimately, one must question whether an improvement in crystal yield, for example, is worth the additional difficulties which may arise during other aspects of design and operation.

To address this question, the general formulation is adjusted to account for four possible scenarios: cooling crystallization in a pure solvent (CP); cooling crystallization in a solvent mixture (CM); antisolvent crystallization at fixed temperature starting with a pure solvent in the initial state (ASP); and antisolvent crystallization at fixed temperature where solvent mixtures are considered in both the initial and final states (ASM). For the cases including cooling crystallization (CP and CM), the maximum allowable temperature is fixed at 373.15 K. Whilst this is also true for the cases involving antisolvent crystallization, it is expected that lower temperatures would provide increased crystal yields due to the final solubility of lovastatin being lower. Due to the additional costs associated with reducing the operating temperature much below that of cooling water, the lower temperature limit is fixed at 293.15 K for all of the design problems.

In all scenarios, solvents (and antisolvents) are selected from a list of thirteen candidates, shortlisted based on their low toxicity and the availability of the necessary interaction parameters with lovastatin within the SAFT-γ Mie framework. Hence, there are 78 unique solvent pairings to select from. Solvent consumption is also investigated, whereby a lower consumption is preferable, but with lower limits of 3.5 g solvent/(g crystal) and 4 mL solvent/(g crystal) to prevent the formation of highly viscous slurries. A summary of these model inputs can be found in Table 1.

**Table 1:** Inputs Required to Describe the CAM^b^D Problem for the Crystallization of Lovastatin, Where Technique-Specific Constraints are Required to Describe Cooling Crystallization or Antisolvent Crystallization.^a^

description	model inputs
number of solvents and API	number of solvents = 13, API = lovastatin
candidate solvents *N*_S_ (78 potential binary solvent pairs)	water, *n*-pentane, *n*-heptane, ethanol, 1-propanol, 1-butanol, 1-pentanol, methyl acetate, ethyl acetate, propyl acetate, isopropyl acetate, butyl acetate, isobutyl acetate
temperature limits	*T*_min_ = 293.15 K, *T*_max_ = 373.15 K
solvent mass limits	χ_s_ ≥ 3.5 g solvent/(g crystal), *V*_s_ ≥ 4.0 mL solvent/(g crystal)
technique-specific constraints	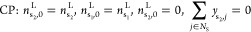
	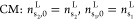
	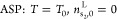
	ASM: *T* = *T*_0_

a Here, s_1_, s_2_, and API refer to the solvent, antisolvent, and lovastatin, respectively. The operating temperatures (*T*_0_ and *T*) are bounded by upper and lower limits, *T*_max_ and *T*_min_, respectively, whilst the solvent mass is constrained only by lower limits, on a mass (χ_s_) and a volumetric (*V*_s_) basis. The moles of solvent ii in the liquid phase are represented by *n*_ii_^L^, with the added subscript 0 to denote the initial state of the system. The sum of binary variables *y*_s_2_,*j*_ represents the presence of an antisolvent and is set to zero in mode CP to ensure a pure solvent is obtained.

A ranked list of optimal solvent blends and process conditions is given in Table 2, where the best solution—that with the highest crystal yield of lovastatin—is given a ranking of 1. In all relevant design problems, the final operating temperature is 293.15 K—the lower temperature limit of the case study. Furthermore, all solutions exist at the limit of one of the implemented constraints—the solvent mass limit (χ_s_ = 3.5 g solvent/(g crystal)), the temperature limits (*T*_0_ = 293.15 K or *T*_0_ = 373.15 K), or the solvent liquid range limit (*T*_0_ = *T*_mix_^b^ – 10)—such that relaxing these constraints may further improve the crystal yield.

**Table 2:** Results of the Optimization of Lovastatin Crystal Yield, *Y*_API_, for Five Crystallization Methods—Cooling Crystallization with: Only Pure Solvent Allowed in the Initial State (CP), Mixtures Allowed Throughout (CM); Antisolvent Crystallization with: Only Pure Solvent Allowed in the Initial State (ASP), Mixtures Allowed Throughout (ASM); and Hybrid Cooling and Antisolvent Crystallization (H)^a^

							solvents	
method	rank	*Y*_API_/%	χ_s_/(g/g)	*T*_0_/K	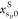	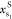	s_1_	s_2_
cooling (CP)	1	97.47	73.02	361.15	1.000	1.000	*n*-heptane	
	2	96.33	3.50	368.75	1.000	1.000	isobutyl acetate	
	3	94.89	3.50	363.35	1.000	1.000	1-pentanol	
	4	93.46	4.11	356.35	1.000	1.000	isopropyl acetate	
	5	92.75	3.50	357.05	1.000	1.000	butyl acetate	
								
cooling (CM)	1	97.47	73.02	361.15	1.000	1.000	*n*-heptane	
	2	96.45	3.50	369.15	0.962	0.962	isobutyl acetate	*n*-pentane
	3	94.89	3.50	363.35	1.000	1.000	1-pentanol	
	4	93.46	4.11	356.35	1.000	1.000	isopropyl acetate	
	5	93.36	3.50	358.25	0.915	0.915	butyl acetate	*n*-pentane
								
anti-solvent (ASP)	1	92.16	85.84	293.15	1.000	0.158	methyl acetate	*n*-heptane
	2	90.24	109.56	293.15	1.000	0.165	ethyl acetate	*n*-heptane
	3	89.85	78.01	293.15	1.000	0.134	methyl acetate	*n*-pentane
	4	87.22	101.97	293.15	1.000	0.139	ethyl acetate	*n*-pentane
	5	85.62	170.49	293.15	1.000	0.174	propyl acetate	*n*-heptane
								
anti-solvent (ASM)	1	93.04	75.80	293.15	0.928	0.157	methyl acetate	*n*-heptane
	2	91.67	63.10	293.15	0.882	0.133	methyl acetate	*n*-pentane
	3	90.24	109.56	293.15	1.000	0.165	ethyl acetate	*n*-heptane
	4	87.40	100.40	293.15	0.955	0.139	ethyl acetate	*n*-pentane
	5	85.62	170.49	293.15	1.000	0.174	propyl acetate	*n*-heptane
								
hybrid (H)	1	99.23	7.93	370.35	1.000	0.174	propyl acetate	*n*-heptane
	2	99.16	8.59	373.15	1.000	0.184	butyl acetate	*n*-heptane
	3	98.97	7.25	370.35	1.000	0.145	propyl acetate	*n*-pentane
	4	98.93	10.88	373.15	1.000	0.228	isobutyl acetate	*n*-heptane
	5	98.90	7.77	373.15	0.954	0.154	butyl acetate	*n*-pentane

a For each method, the top five solvent mixtures are ranked with respect to the crystal yield of lovastatin achieved. The solvent consumption, χ_s_, initial temperature, *T*_0_, and binary solvent compositions in terms of initial and final mole fractions of solvent s_1_,  and , are also given. In all cases, the maximum allowable temperature is 373.15 K, the minimum solvent mass is 3.5 g solvent/(g crystal), and the final temperature is 293.15 K.

Comparing the two modes of cooling crystallization, CP and CM, it can be observed that crystallizing lovastatin from pure *n*-heptane is predicted to provide the highest crystal yield. However, due to the low solubility of lovastatin in *n*-heptane at the maximum allowable temperature, the solvent consumption is also much higher than in many other solvents—a clear example of the merit of reviewing both crystal yield and solvent consumption when looking for optimal solvent blends. In CP mode, three of the remaining top five solvents, isobutyl acetate, isopropyl acetate, and butyl acetate, are constrained by the lower limit on solvent use—implemented to prevent the formation of highly viscous slurries—and therefore the entire temperature range cannot be utilized for cooling. Such solutions to the optimization problem would not be feasible had the temperature range been fixed *a priori*. For the other two solutions, *n*-heptane and 1-pentanol, the solvent phase limits are active at the solvent boiling point, and thus, the initial temperature could not feasibly be increased further. Using the general formulation again allows these volatile solvents to be found as solutions, rather than removing them based on the boiling point during a prescreening step.

Allowing the cooling crystallization to utilize solvent mixtures (CM mode) results in similar solutions, with the same selection of top tier solvents as those found in the CP mode. However, for solutions ranked 2 and 5, the crystal yield is increased by introducing a small amount of *n*-pentane into the mixture. Although this reduces the solubility of lovastatin in the initial state, meaning the mass of solvent required to solubilize the same mass of lovastatin is greater than in the CP mode, it also provides a means for the final solubility of lovastatin to be lower. The higher initial temperature thus maintains the low solvent use. The combination of these two effects leads to an increase in the crystal yield, maintaining the solvent use at the lower limit of 3.5 g solvent/(g crystal).

The top five optimal solvent blends for both modes of operation for antisolvent crystallization, ASP and ASM, are identical, although their specific rankings are different depending on how the crystallizer is operated. Three of the solvent mixtures lead to higher crystal yields when utilizing a solvent mixture in the initial state, also leading to a decrease in solvent consumption. As such, the blend consisting of methyl acetate and *n*-pentane is ranked second in ASM mode, compared to third in ASP mode. This blend replaces ethyl acetate and *n*-heptane at rank 2, for which an initial state in pure ethyl acetate is optimal. As expected, the final process temperature is minimized to reduce the solubility of lovastatin and is therefore 293.15 K in all ten cases. For both modes of operation, the optimal crystal yields achieved are lower than those found when employing cooling crystallization. Generally, the solvent consumption for antisolvent crystallization is an order of magnitude greater than that of cooling crystallization, although it should be noted that no upper constraint has been imposed on solvent use.

In the scenarios considering the hybrid technique (H), it is seen that combinations of acetates and *n*-heptane or *n*-pentane, where the latter are added as antisolvent, are found to provide the highest crystal yields. All five solutions utilize both cooling and antisolvent crystallization to achieve a significantly greater crystal yield of lovastatin than any of the solutions based on standard techniques. Furthermore, solvent mixtures that include propyl acetate as the operating solvent cannot be operated at the maximum temperature specified due to the lower bubble point of the mixture (the constraint on the solvent liquid range); previous CAM^b^D approaches would have screened out this optimal result.

Ultimately, it is predicted that the hybrid technique can significantly outperform antisolvent crystallization of lovastatin, whilst also achieving a higher crystal yield than cooling crystallization alone. Our study suggests that the use of hybrid cooling and antisolvent crystallization would be beneficial in the production of lovastatin. Recrystallisation in acetone and water mixtures is typically employed^39^ as a purification technique in industry, although studies into the use of alcohols and homologous acetates have also been considered.^40^ Whilst this work considers the thermodynamic effects of the choice of solvent on the crystallization system, the crystal habit can also influence this decision, due to the propensity of lovastatin to form undesirable needle-like crystals. The consideration of habit is however beyond the scope of our current paper and will be the focus of further research.

#### Effect of Operating Temperature on the Optimal Solvent Blend

It is apparent from Table 2 that several optimal solutions result in an initial process temperature that is lower than the maximum temperature specified, suggesting that there are effective but volatile solvent blends that can provide a better performance at lower temperatures, as hypothesized. To assess further the importance of operating temperature on the outcome of the crystallization solvent design problem, eight design scenarios are posed; a different upper temperature limit is used in each, ranging between 303.15 and 373.15 K. As before, the lower temperature limit is fixed at 293.15 K. Solvents (and antisolvents) are selected from the same shortlist of thirteen solvent candidates as before. For each design problem, a list of the most optimal solvent blends is generated, ranked by the respective crystal yield of lovastatin (refer to Table 3). Again, lower limits of 3.5 g solvent/(g crystal) and 4 mL solvent/(g crystal) are imposed on the solvent consumption.

**Table 3:** Results of the Optimization of Lovastatin Crystal Yield, *Y*_API_, for Eight Scenarios with Different Maximum Temperature Limits^a^

							solvents	
*T*_max_/K	rank	*Y*_API_/%	χ_s_/(g/g)	*T*_0_/K	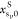	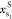	s_1_	s_2_
303.15	1	95.31	49.62	303.15	0.932	0.158	methyl acetate	*n*-heptane
	2	94.31	41.93	303.15	0.907	0.133	methyl acetate	*n*-pentane
	3	93.57	69.61	303.15	1.000	0.165	ethyl acetate	*n*-heptane
	4	91.67	63.40	303.15	0.957	0.139	ethyl acetate	*n*-pentane
								
313.15	1	96.75	33.91	313.15	0.934	0.158	methyl acetate	*n*-heptane
	2	95.66	31.54	313.15	0.984	0.133	methyl acetate	*n*-pentane
	3	95.65	46.00	313.15	1.000	0.165	ethyl acetate	*n*-heptane
	4	94.37	41.66	313.15	0.957	0.139	ethyl acetate	*n*-pentane
								
323.15	1	97.05	30.65	315.95	0.936	0.158	methyl acetate	*n*-heptane
	2	96.99	31.41	323.15	1.000	0.165	ethyl acetate	*n*-heptane
	3	96.11	28.28	323.15	0.950	0.139	ethyl acetate	*n*-pentane
	4	96.02	28.77	316.45	1.000	0.133	methyl acetate	*n*-pentane
								
333.15	1	97.87	22.04	333.15	1.000	0.165	ethyl acetate	*n*-heptane
	2	97.25	19.75	333.15	0.962	0.139	ethyl acetate	*n*-pentane
	3	97.16	29.70	333.15	1.000	0.174	propyl acetate	*n*-heptane
	4	97.05	30.65	315.95	0.936	0.158	methyl acetate	*n*-heptane
								
343.15	1	98.44	16.00	342.75	1.000	0.165	ethyl acetate	*n*-heptane
	2	98.03	20.42	343.15	1.000	0.174	propyl acetate	*n*-heptane
	3	97.96	14.51	342.75	1.000	0.139	ethyl acetate	*n*-pentane
	4	97.40	18.70	343.15	0.972	0.145	propyl acetate	*n*-pentane
								
353.15	1	98.61	14.27	353.15	1.000	0.174	propyl acetate	*n*-heptane
	2	98.44	16.00	342.75	1.000	0.165	ethyl acetate	*n*-heptane
	3	98.21	18.50	353.15	1.000	0.184	butyl acetate	*n*-heptane
	4	98.18	12.96	353.15	0.954	0.145	propyl acetate	*n*-pentane
								
363.15	1	99.01	10.11	363.15	1.000	0.174	propyl acetate	*n*-heptane
	2	98.78	12.57	363.15	1.000	0.184	butyl acetate	*n*-heptane
	3	98.70	9.19	363.15	0.983	0.145	propyl acetate	*n*-pentane
	4	98.44	16.00	342.75	1.000	0.165	ethyl acetate	*n*-heptane
								
373.15	1	99.23	7.93	370.35	1.000	0.174	propyl acetate	*n*-heptane
	2	99.16	8.59	373.15	1.000	0.184	butyl acetate	*n*-heptane
	3	98.97	7.25	370.35	1.000	0.145	propyl acetate	*n*-pentane
	4	98.93	10.88	373.15	1.000	0.228	isobutylacetate	*n*-heptane

a For each method, the top four solvent mixtures are ranked with respect to the crystal yield of lovastatin achieved. The solvent consumption, χ_s_, initial temperature, *T*_0_, and binary solvent composition, , are also given. In all cases, the minimum solvent consumption is 3.5 g/g, and the final temperature is 293.15 K.

**Figure 4:**
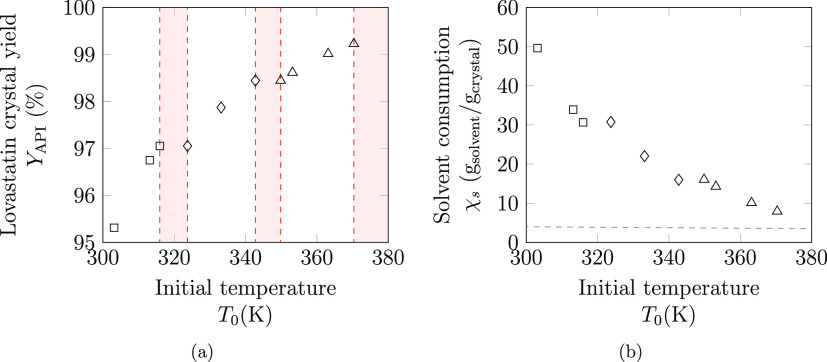
Results of the optimization of lovastatin crystal yield using hybrid crystallization, for different maximum operating temperatures, between 303.15 and 373.15 K in 10 K increments. The lower bound on the final temperature for all crystallizations is 293.15 K, whilst the upper temperature limit is changed for each design problem, which in turn affects the initial operating temperature shown in the figure. The optimal solvent is indicated with the symbols □, ◊, and △, referring to methyl acetate, ethyl acetate, and propyl acetate, respectively. In all cases, the optimal antisolvent is *n*-heptane. (a) Maximum crystal yield and optimal initial temperatures. In most cases, the optimal initial temperature is at the temperature upper bound but any crystallization operating at an initial temperature within the corresponding red shaded region is predicted to achieve a lower crystal yield relative to the designs on the vertical dashed lines. (b) The solvent consumption is shown for each optimal solvent mixture (that which maximizes the crystal yield) over the temperature range, where the horizontal dashed line is the lower solvent mass limit, 3.5 g solvent/(g crystal).

As represented in Figure 4, the combination of cooling and antisolvent crystallization leads to high crystal yields for all eight scenarios considered, utilizing solvent mixtures throughout. As expected, relaxing the upper temperature limit—that is, allowing the crystallization to operate at higher initial temperatures—*generally* leads to a higher crystal yield. Increasing the upper temperature limit often leads to a change in the solvent mixture ranking, as more volatile solvents cannot be utilized at the higher initial temperatures. Furthermore, extending the temperature range leads to a decrease in the overall solvent consumption of crystallization. This is due to the higher solubility of lovastatin at higher temperatures, meaning less solvent is required to completely solubilize the API initially.

It is, however, not always optimal to begin the crystallization at the highest allowable temperature. As depicted in Figure 4, any crystallization operating at an initial temperature within a red shaded region is predicted to achieve a lower crystal yield than either of the optimal solutions on the boundaries of the region (the vertical dashed lines). Thus, the red shaded regions indicate suboptimal temperatures. For instance, in Table 3, four design scenarios result in mixtures of methyl acetate and *n*-heptane being one of the top four most optimal solvent blends; for three of these cases, this outcome is the highest ranked optimal solution. Accounting for the temperature buffer of 10 K, the combination of methyl acetate and *n*-heptane cannot be used at temperatures above 315.95 K because the constraint on the liquid range of the solvent would be violated (*T*_mix_^b^ = 325.95 K). For the first two design scenarios (*T*_max_ of 303.15 and 313.15 K), methyl acetate and *n*-heptane are the optimal solvent blend, and the crystallization can be operated at the maximum temperature limit (*T*_0_ = *T*_max_ < *T*_mix_^b^ – 10). However, when the maximum allowable temperature is fixed at 323.15 K, the optimal solution (*Y*_API_ = 97.05%) is again found to be a blend of methyl acetate and *n*-heptane with an initial temperature of only 315.95 K, as the next best mixture (ethyl acetate and *n*-heptane) yields a lower yield (*Y*_API_ = 96.99%) despite being able to utilize a further 7.2 K of cooling.

This effect is replicated when the maximum temperature limit is fixed at 343.15 K, where it is found that the optimal result is a mixture of ethyl acetate and *n*-heptane, with an initial operating temperature of 342.75 K, and for the design case with an upper temperature limit of 373.15 K, for which propyl acetate and *n*-heptane produce an optimal mixture with an initial operating temperature of 370.35 K.

Examining the 5 top solutions for each of the eight design scenarios, there are nine solutions that operate at initial temperatures below the upper temperature limit. Clearly, maximizing the operating temperature range, such that *T*_0_ – *T* = *T*_max_ – *T*_min_, is not always optimal, and this emphasizes the importance of using a general formulation in which molecular and process decisions are optimized simultaneously. Furthermore, the bubble points of many of the optimal mixtures are greater than the normal boiling point of the most volatile solvent in each blend, highlighting the advantage of considering the bubble temperature of the mixture rather than the boiling temperatures of the pure components in such designs.

From a practical perspective, the optimal values returned by the solver are very precise and this precision could not be achieved in practice. By rounding some of the solutions (*e.g.*, hybrid solutions 1 and 2 in Table 3) to the nearest 5 K in temperature and to one significant figure in mole fraction, we find only a mild effect on the predicted yield and no change in ranking. A key conclusion is thus that there are several solvent/process combinations that give very high yields, albeit with varying levels of solvent consumption.

#### Reducing Solvent Consumption during Crystallization

Excessive solvent use in industrial processes is a well-recognized problem, with solvents typically accounting for more than 80% of process mass^41^ and the majority of energy use^42^ in the production of pharmaceuticals. Whilst efforts are being made to include and improve solvent recovery systems, process intensification is a crucial step toward a “greener” manufacturing process. In addition, reducing the volume of solvent required on-site can improve the inherent safety of the facility. As such, it is important to design crystallization processes that are not only efficient in terms of the API crystal yield, but also with regards to solvent consumption. To understand the relationship between crystal yield and solvent use, a multiobjective optimization formulation is solved, with the simultaneous objectives of maximizing crystal yield and minimizing solvent consumption, on a mass basis. This is solved using the ϵ-constraint method, with ten values of ϵ, so that ten instances of the single-objective, yield-maximization, optimization problem are solved with a different maximum allowable solvent consumption imposed for each instance. It should be noted that the lower solvent consumption limits remain fixed at 3.5 g solvent/(g crystal) and 4 mL solvent/(g crystal). The same list of thirteen solvents is available for design, and the upper and lower temperature limits are fixed at 373.15 and 293.15 K, respectively.

**Figure 5:**
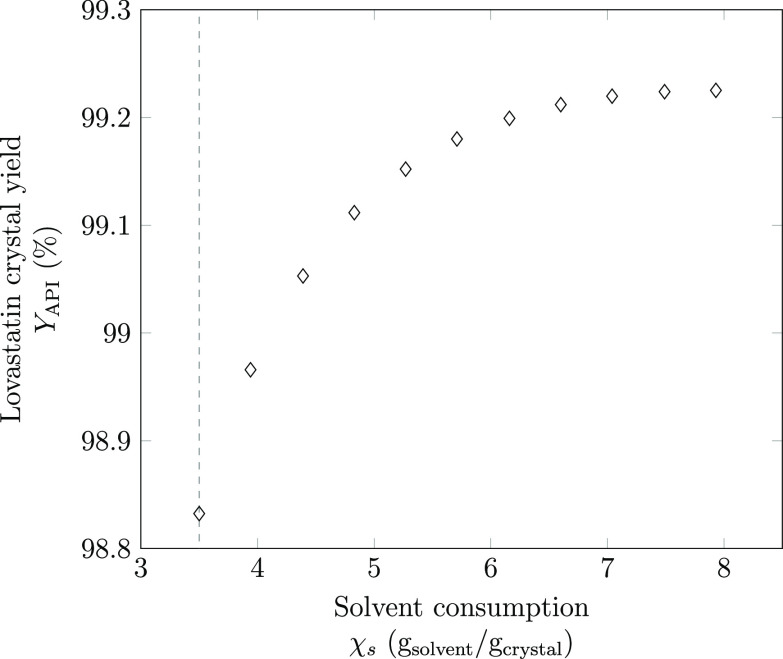
Pareto front for the crystal yield of lovastatin and solvent consumption, where χ_s_ = 7.93 g solvent/(g crystal) corresponds to an unconstrained solvent consumption. All of the Pareto points have an optimal initial temperature of 370.35 K and final temperature of 293.15 K, for lovastatin in a mixture of propyl acetate and *n*-heptane. The vertical dashed line is the lower solvent mass limit, 3.5 g solvent/(g crystal).

For all ten resulting Pareto points, the optimal solvent mixtures are found to be blends of propyl acetate and *n*-heptane in varying proportions (Table 4). It is clear from Figure 5 that there is a trade-off between achieving a high crystal yield and a low consumption of solvent. From these results, it is possible to choose a compromise solution, whereby less solvent is used during the crystallization, but a high crystal yield is still attained.

**Table 4:** Results of the Multiobjective Optimization of Lovastatin Crystal Yield and Solvent Consumption, χ_s_max__^a^

						solvents	
χ_s_max__/(g/g)	*Y*_API_/%	χ_s_/(g/g)	*T*_0_/K	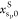	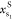	s_1_	s_2_
Unconstrained	99.23	7.93	370.35	1.000	0.174	propyl acetate	*n*-heptane
7.49	99.22	7.49	370.35	1.000	0.184	propyl acetate	*n*-heptane
7.04	99.22	7.04	370.35	1.000	0.196	propyl acetate	*n*-heptane
6.60	99.21	6.60	370.35	1.000	0.209	propyl acetate	*n*-heptane
6.16	99.20	6.16	370.35	1.000	0.225	propyl acetate	*n*-heptane
5.71	99.18	5.71	370.35	1.000	0.242	propyl acetate	*n*-heptane
5.27	99.15	5.27	370.35	1.000	0.263	propyl acetate	*n*-heptane
4.83	99.11	4.83	370.35	1.000	0.287	propyl acetate	*n*-heptane
4.39	99.05	4.39	370.35	1.000	0.316	propyl acetate	*n*-heptane
3.94	98.97	3.94	370.35	1.000	0.352	propyl acetate	*n*-heptane
3.50	98.83	3.50	370.35	1.000	0.398	propyl acetate	*n*-heptane

a The solvent consumption, χ_s_, initial temperature, *T*_0_, and binary solvent composition, as initial and final mole fractions of s_1_,  and , are also given. In all cases, the minimum solvent mass is 3.5 g solvent/(g crystal), the upper temperature limit is 373.15 K and the final temperature is 293.15 K.

When the solvent consumption is unconstrained, the maximum crystal yield is found to be 99.23% and the corresponding solvent use is 7.93 g solvent/(g crystal). As the maximum allowable solvent consumption is reduced *via* an ϵ-constraint, the optimal amount of antisolvents to be added to the process decreases. This has a detrimental impact on the API crystal yield as the mole fraction of antisolvent, *n*-heptane, in the final solvent mixture is reduced and the final solubility of lovastatin is thus increased. The initial conditions of the crystallization, however, remain unchanged in all Pareto points. These conditions correspond to the highest possible solubility of API in the initial solvent mixture and hence the lowest mass of solvent required to solubilize a given mass of lovastatin.

### Case Study II: Crystallization of Ibuprofen

**Figure 6:**
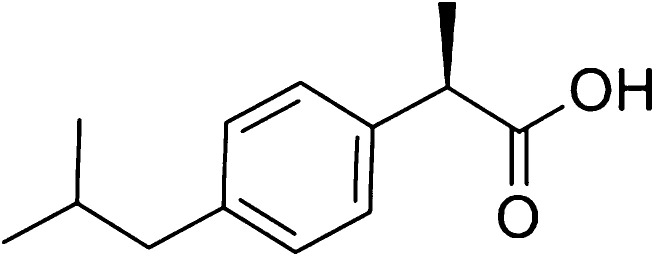
Chemical structure of ibuprofen, the API considered in Case Study II.

Ibuprofen is a nonsteroidal anti-inflammatory drug (NSAID), widely used around the world to manage pain. Whilst the crystallization of this API has already been thoroughly researched in both academia^24,43,44^ and industry,^45−47^ with global production estimated to be greater than 35,000 tonnes per annum,^48^ ibuprofen remains an interesting compound to study in the general crystallization solvent design problem. It is well documented that ibuprofen crystallizes by forming dimers around the carboxylic acid functional groups due to hydrogen bonding^49^ (Figure 6); the presence of dimers in solution can be modeled with the SAFT-γ Mie group-contribution approach^26^*via* the introduction of association sites on the carboxyl functional group,^27^ accurately predicting the solubility of ibuprofen in organic solvents. Specifically, the root mean squared error in the logarithm (log_10_) of the solubility of ibuprofen is found to be 0.12 log units when experimental data in acetone, *n*-butanol, water, and *n*-heptane are compared to the predicted values obtained with the model used in our current work,^27,30^ over a range of temperatures. The predictions are especially accurate in water, acetone, and *n*-butanol, while the solubility of ibuprofen in *n*-heptane is underestimated. In the presence of solvent molecules, there can be competition between the formation of dimers and the formation of hydrogen bonds between the carboxylic group and solvent molecules, making it important to rely on quantitative predictions of the solubility in crystallization process design.

The objective of the design problem considered here is to identify the optimal solvent blend and process conditions that maximize the crystal yield of ibuprofen. For all scenarios investigated, the solvents (and antisolvents) are selected from eight candidate molecules, short-listed based on their low toxicity and the availability of the necessary interaction parameters in the SAFT-γ Mie framework. As such, there are 28 potential binary solvent pairs to select from during the optimization. For all cases studied, the lower temperature limit is fixed at 293.15 K, whilst the lower limits of solvent consumption are set at 3.5 g_solvent_/(g_crystal_) and 4 mL_solvent_/(g_crystal_). A summary of these model inputs can be found in Table 5.

**Table 5:** Inputs Required to Describe the CAM^b^D Problem for the Crystallization of Ibuprofen, Where Technique-Specific Constraints are Required to Describe Cooling Crystallization or Antisolvent Crystallization^a^

description	model inputs
number of solvents and API	number of solvents = 8; API = ibuprofen
candidate solvents *N*_S_ (28 potential binary solvent pairs)	water, *n*-pentane, *n*-heptane, ethanol, 1-propanol, 1-butanol, 1-pentanol, acetone
temperature limits	*T*_min_ = 293.15 K, *T*_max_ = 318.15 K
solvent mass limits	χ_s_ ≥ 3.5 g_solvent_ g_crystal_^–1^, *V*_s_ ≥ 4.0 mL_solvent_ g_crystal_^–1^
technique-specific constraints	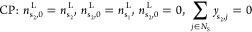
	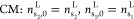
	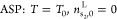
	ASM: *T* = *T*_0_

a Here, s_1_, s_2_, and API refer to the solvent, the antisolvent, and ibuprofen, respectively. The operating temperatures (*T*_0_ and *T*) are bounded by upper and lower limits, *T*_max_ and *T*_min_, respectively, whilst the solvent mass is constrained only by lower limits, on a mass (χ_s_) basis and on a volumetric (*V*_s_) basis. The final (initial) moles of solvent ii in the liquid phase are represented by *n*_ii_^L^ (*n*_ii,0_^L^). The binary variables *y*_s_2_,*j*_ express the selection of a candidate antisolvent (from the list *N*_S_) and are included here to prevent the choice of antisolvent being a design decision in crystallization mode CP.

#### Comparison of Conventional Approaches and Hybrid Crystallization

As in the case study for lovastatin, it is important to compare the hybrid approach to the standard modes of operation—cooling or antisolvent crystallization operating independently. As such, five design scenarios are considered: cooling crystallization in a pure solvent (CP); cooling crystallization allowing solvent mixtures (CM); antisolvent crystallization where ibuprofen is initially dissolved in a pure solvent (ASP); antisolvent crystallization where solvent mixtures can be used throughout (ASM); and the hybrid technique (H). In each of these scenarios, the top four optimal solvent blends are ranked based on the crystal yield obtained, and the maximum allowable temperature is fixed at 318.15 K. In all solutions, the final temperature is found to be 293.15 K—the minimum allowable temperature.

As shown in Table 6, for the design scenario considering cooling crystallization from a pure solvent (CP), *n*-heptane is found to provide the best performance. The second-ranked solvent is found to be water; due to ibuprofen being virtually insoluble in water, the solvent consumption is very high and the result is highly impractical. Crystal yields below 50% are obtained in the remaining two solvents (1-pentanol and *n*-pentane), where the complete temperature range cannot be utilized because of either the lower limit on solvent consumption on a mass basis (1-pentanol) or the solvent liquid range constraint (*n*-pentane). Overall, these results suggest that only one of the eight solvents chosen is applicable to cooling crystallization.

**Table 6:** Results of the Optimization of Ibuprofen Crystal Yield, *Y*_API_, for Five Crystallization Methods—Cooling Crystallization with: Only Pure Solvent Allowed in the Initial State (CP), Mixtures Allowed Throughout (CM); Antisolvent Crystallization with: Only Pure Solvent Allowed in the Initial State (ASP), Mixtures Allowed Throughout (ASM), and Hybrid Cooling and Antisolvent Crystallization (H)^a^

							solvents	
method	rank	*Y*_API_/%	χ_s_/(g/g)	*T*_0_/K	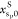	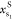	s_1_	s_2_
cooling (CP)	1	87.18	11.02	318.15	1.000	1.000	*n*-heptane	
	2	78.96	3498.90	318.15	1.000	1.000	water	
	3	44.97	3.50	307.65	1.000	1.000	1-pentanol	
	4	35.41	55.49	298.65	1.000	1.000	*n*-pentane	
								
cooling (CM)	1	90.07	3.50	318.15	0.133	0.133	ethanol	*n*-heptane
	2	90.00	3.50	318.15	0.147	0.147	1-propanol	*n*-heptane
	3	89.90	3.50	318.15	0.166	0.166	1-butanol	*n*-heptane
	4	89.77	3.50	318.15	0.191	0.191	1-pentanol	*n*-heptane
								
anti-solvent (ASP)	1	99.83	8.64	293.15	1.000	0.040	ethanol	water
	2	99.78	10.77	293.15	1.000	0.027	acetone	water
	3	99.71	14.42	293.15	1.000	0.027	1-propanol	water
	4	99.51	23.74	293.15	1.000	0.020	1-butanol	water
								
anti-solvent (ASM)	1	99.83	8.64	293.15	1.000	0.040	ethanol	water
	2	99.81	9.19	293.15	0.641	0.027	acetone	water
	3	99.71	14.42	293.15	1.000	0.027	1-propanol	water
	4	99.52	23.25	293.15	0.624	0.020	1-butanol	water
								
hybrid (H)	*1*	*99.94*	*4.00*	*318.15*	*0.475*	*0.021*	*acetone*	*water*
	2	99.92	4.09	318.15	0.517	0.040	ethanol	water
	*3*	*99.90*	*4.80*	*318.15*	*0.423*	*0.027*	*1-propanol*	*water*
	*4*	*99.88*	*5.78*	*318.15*	*0.438*	*0.020*	*1-butanol*	*water*

a For each method, the top four solvent mixtures are ranked with respect to the crystal yield of ibuprofen achieved. Solvent consumption, χ_s_, initial temperature, *T*_0_, and binary solvent composition, , are also given. In all cases, the maximum allowable temperature is 318.15 K, the minimum solvent mass is 3.5 g solvent/(g crystal), and the final temperature is 293.15 K. Italicized solutions are optimal mixtures with additional composition constraints, to prevent the occurrence of LLE.

**Figure 7:**
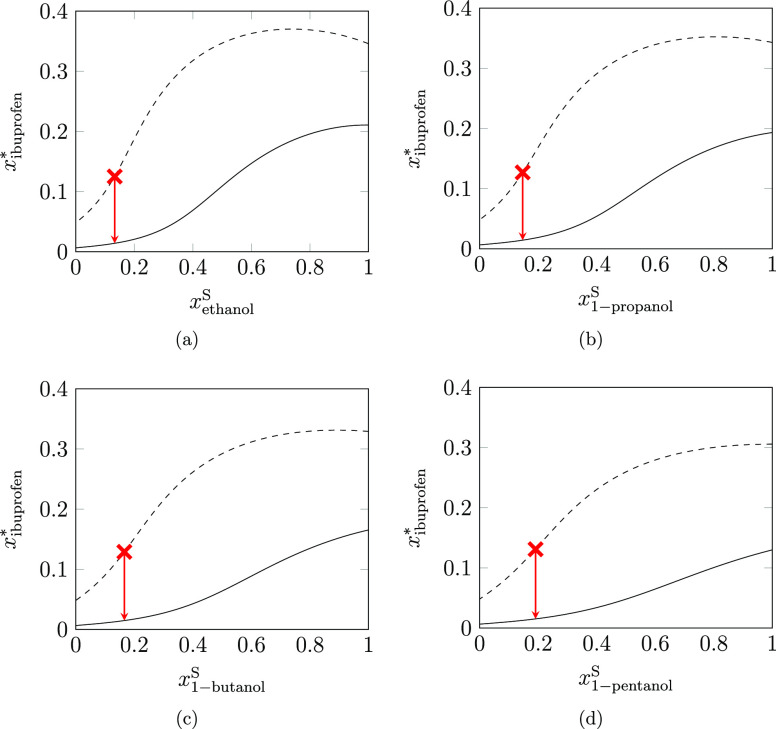
Predicted solubility of ibuprofen, *x*_ibuprofen_^*^, as a function of the mole fraction of alcohol in binary solvent mixtures of primary alcohols and *n*-heptane at 293.15 K (solid curve) and 318.15 K (dashed curve): (a) ethanol, (b) 1-propanol, (c) 1-butanol, and (d) 1-pentanol. In mode CM, the mixture marked with a red cross is cooled, reducing the solubility of ibuprofen as indicated by the red arrows. The solubilities are calculated using the SAFT-γ Mie group-contribution approach in gPROMS.

However, expanding the design space to include solvent mixtures (CM) improves the usefulness of the primary alcohols; the combination of primary alcohols with *n*-heptane leads to an absolute increase in crystal yield of at least 2.5%, whilst achieving the minimum allowable solvent consumption for the process. Taking the case of ibuprofen dissolved in an *n*-heptane-rich mixture with a primary alcohol, it is clear from Figure 7 that the solubility of ibuprofen increases at a much greater rate, relative to the composition of alcohol, at higher temperatures. Hence, by blending small proportions of the selected alcohol with *n*-heptane, a higher initial solubility is achieved relative to pure *n*-heptane, reducing the mass of the solvent required to completely dissolve ibuprofen. Upon cooling the solution to 293.15 K, the high levels of *n*-heptane in the mixture cause a significant drop in the solubility of ibuprofen, leading to a higher crystal yield. The merit of using solvent mixtures in the cooling crystallization framework is evident in both improving crystal yield and reducing solvent consumption in the crystallization of ibuprofen.

The two design scenarios considering antisolvent crystallization, ASP and ASM, differ only marginally in results, producing very high crystal yields. In all eight solutions, water is selected as the optimal antisolvent, whilst the operating temperature is fixed at the lower limit of 293.15 K. Whilst allowing the use of solvent mixtures throughout the crystallization (mode ASM) can improve the crystal yield and reduce solvent consumption in two of the top-ranked cases, the change is minimal.

Similar to the results for lovastatin, the solutions for the design scenario considering the hybrid technique (H) show that the combined use of cooling and antisolvent crystallization leads to greater crystal yields of ibuprofen compared to any of the standard techniques. Furthermore, solvent mixtures are utilized in all stages of operation, highlighting the benefit of employing the hybrid technique. In comparison to the results for the cooling crystallization design problems (modes CP and CM), using the hybrid technique achieves a crystal yield which is more than 11% higher, whilst also maintaining a low solvent consumption. Although the crystal yield of mode H is fractionally higher compared to the antisolvent designs (modes ASP and ASM), and the solvent consumption is between two and five times lower, this difference may not warrant the use of a more complicated hybrid system. Nevertheless, being able to investigate these different modes of operation allows the rapid planning of experimental campaigns, eliminating poor-performing crystallization techniques before time and material are spent testing them.

It should be noted that three of the solvent mixtures used with the hybrid technique (H) are initially found to exhibit liquid–liquid equilibrium; these pairs are identified by italics in Table 6. As a result, additional constraints (eqs 22 and 23) have been included on the binary solvent composition. For each of the three pairs of binary solvents, a different constraint, derived from the stability limits of this specific pair, is used.

#### Effect of Operating Temperature on the Optimal Solvent Blend

Although the crystal yield of the four top-ranked optimal solutions are very similar, it is important to understand the effect of operating temperature on the design of the crystallization solvent blend. As such, five design scenarios are posed; in each, the upper temperature limit is different, ranging between 298.15 and 318.15 K, and the lower temperature limit is fixed at 293.15 K. Solvent consumption is also examined, whereby a lower consumption is preferable, but the lower limits are set at 3.5 g_solvent_/(g_crystal_) and 4 mL_solvent_/(g_crystal_). It should also be noted that several of the solutions of the initial optimization problem are found to exhibit LLE when considering the ternary solvent-antisolvent-API mixture—this is even the case for mixtures containing acetone and water, which are miscible in all proportions for the given temperature range, but where the presence of ibuprofen induces phase separation. Once again, additional constraints are placed upon the optimization problem for those cases, as described in eqs 22 and 23; this has a negligible impact, and does not result in a change in the ranking of solutions with only a decrease in crystal yield by less than 0.01% in absolute terms. However, it is worth noting that the miscibility limits on solvent composition are active in these solutions, and thus, small perturbations may result in an unstable mixture. As such, an additional buffer-zone could be included in eq 22 to move the operating conditions sufficiently outside the LLE envelope.

**Figure 8:**
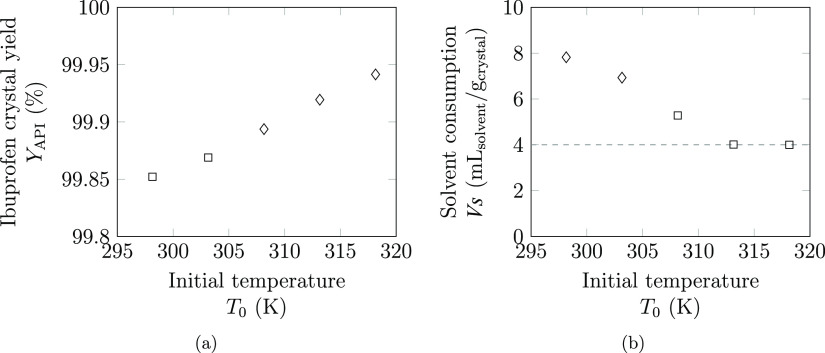
Results of the optimization of the ibuprofen crystal yield for different operating temperatures. The final temperature for all crystallizations is fixed at 293.15 K, whilst the upper temperature limit is changed for each design, which in turn affects the initial operating temperature. The optimal solvents, ethanol and acetone, are indicated by the symbols □ and ◊, respectively. In all cases, the optimal antisolvent is water. (a) The maximum crystal yield is expressed for initial temperatures between 298.15 and 318.15 K. (b) The solvent consumption is shown for each optimal solvent mixture (the mixture which maximizes the crystal yield) over the temperature range, where the horizontal dashed line is the lower solvent volume limit, 4.0 mL/g.

As represented in Figure 8, combined cooling and antisolvent crystallization is an effective way to use solvent mixtures, leading to a very high crystal yield, which is only marginally improved by relaxing the upper temperature limit; from the results presented in Table 6 it is evident that the principal driving force is from the addition of water as an antisolvent. In contrast to the case of lovastatin, there are no areas of suboptimal temperatures for this crystallization (no red shaded regions as in Figure 2); it is important to note that within the range of operating temperatures considered here, none of the four most optimal solvent blends are at their respective liquid range limit; all four blends are feasible across the entire temperature range. Despite this, the optimal solvent changes from ethanol to acetone at temperatures higher than 303.15 K. This highlights that it is crucial not to extrapolate results from one operating temperature to another, even for temperatures that lie within the liquid range of the solvent.

Relaxing the upper temperature limit may have a limited impact on the crystal yield in this case, but it leads to significant improvements in the overall solvent consumption. One reason for this is made apparent from the relationship between solubility and temperature—increasing the upper temperature limit makes it possible to employ a higher initial operating temperature, meaning less solvent is required to completely dissolve the ibuprofen initially. However, there is a second effect that compounds this benefit and only arises due to the use of solvent mixtures.

**Figure 9:**
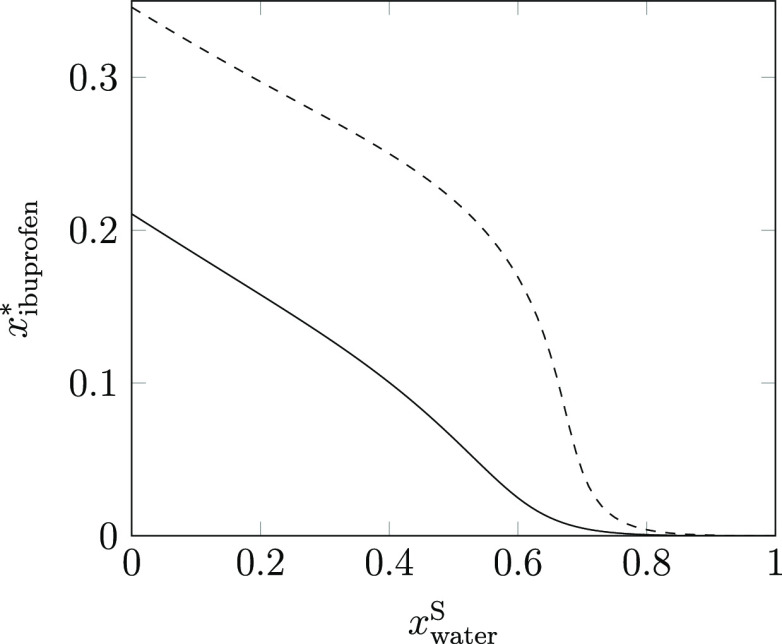
Predicted solubility of ibuprofen, *x*_ibuprofen_^*^, on a molar basis, in mixtures of ethanol and water at 293.15 K (solid line) and 318.15 K (dashed line), calculated using the SAFT-γ Mie group-contribution approach in gPROMS. *x*_water_^S^ refers to the mole fraction of water in the binary solvent mixture.

In Table 7, the initial mole fraction of solvent in the binary solvent mixture is listed for each solvent design problem; the higher the initial temperature, the lower the initial composition of solvent s_1_ in the crystallizer and the higher the initial composition of antisolvent s_2_. It should be noted that the definition of antisolvent here is based on the solubility of ibuprofen in the pure solvent, rather than the solvent mixture; ibuprofen is practically insoluble in pure water, but mixing water with other solvents can significantly increase the solubility of the API relative to the pure solvent. By studying the solubility curves in Figure 9, it is seen that an increase in operating temperature leads to a relative shift of the solubility curve, such that the greatest drop in ibuprofen solubility occurs at a higher composition of antisolvent. Though at low temperatures the effect of unfavorable interactions between the API and antisolvent is so great that even a small addition of water leads to a considerable drop in solubility, at high temperatures these effects are reduced; the system requires considerably more water to reduce the solubility of ibuprofen significantly. As such, at higher initial temperatures, more antisolvents can be present in the initial mixture without negatively impacting the initial solubility of API, and therefore, less additional water is required to achieve the final, low-solubility composition at 293.15 K. It is clear that the integration of cooling and antisolvent crystallization techniques is critical in allowing nonlinear interactions such as these to occur. Such gains are not observed for all APIs; this phenomenon is not present in the case of lovastatin, where the solubility curve shifts in the opposite direction when the temperature is increased. Given the range of possible behaviors, the proposed general approach provides the opportunity for optimal scenarios to be discovered. Indeed, the differences between the two APIs considered highlight the key benefits of CAM^b^D and the importance of applying a general approach to solvent design.

**Table 7:** Comparison of Ranked Solvent Blends for Integrated Cooling and Antisolvent Crystallization of Ibuprofen^a^

						solvents	
*T*_0_/K	rank	*Y*_API_/%	*V*_s_/(mL/g)	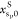	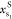	s_1_	s_2_
298.15	1	99.85	7.82	1.000	0.040	ethanol	water
	*2*	*99.84*	*7.90*	*0.603*	*0.027*	*acetone*	*water*
	3	99.74	12.72	1.000	0.027	1-propanol	water
	4	99.61	18.72	0.556	0.020	1-butanol	water
							
303.15	1	99.87	6.93	1.000	0.040	ethanol	water
	*2*	*99.87*	*6.57*	*0.580*	*0.027*	*acetone*	*water*
	3	99.77	11.28	0.679	0.027	1-propanol	water
	4	99.70	14.65	0.497	0.020	1-butanol	water
							
308.15	*1*	*99.89*	*5.28*	*0.553*	*0.027*	*acetone*	*water*
	2	99.88	6.07	1.000	0.040	ethanol	water
	3	99.82	9.09	0.559	0.027	1-propanol	water
	*4*	*99.77*	*11.07*	*0.486*	*0.020*	*1-butanol*	*water*
							
313.15	*1*	*99.92*	*4.01*	*0.519*	*0.027*	*acetone*	*water*
	2	99.90	5.23	1.000	0.040	ethanol	water
	3	99.86	6.92	0.471	0.027	1-propanol	water
	*4*	*99.84*	*7.92*	*0.467*	*0.020*	*1-butanol*	*water*
							
318.15	*1*	*99.94*	*4.00*	*0.475*	*0.021*	*acetone*	*water*
	*2*	99.92	4.13	0.517	0.040	ethanol	water
	3	*99.90*	*4.80*	*0.423*	*0.027*	*1-propanol*	*water*
	*4*	*99.88*	*5.78*	*0.438*	*0.020*	*1-butanol*	*water*

a In all cases, *T*_0_ = *T*_max_, and the final temperature is 293.15 K. Italicized solutions are optimal mixtures with additional composition constraints, to account for the presence of LLE.

## Conclusions

A general formulation has been presented based on a CAM^b^D framework, with an aim to guide experiments toward the identification of optimal solvent blends for the crystallization of pharmaceutical compounds, reducing the time and costs typically associated with solvent selection. By using this general approach, the optimal solvent and antisolvent molecules, their compositions, and the process temperatures required to maximize the crystal yield of a given API can be identified simultaneously, for both the initial and final states of crystallization.

The general formulation has been applied successfully to the crystallization of lovastatin, where optimal solvent mixtures are determined and ranked for several operating temperature ranges, process conditions, and crystallization techniques. Furthermore, we show that constraining the mass of solvent utilized during crystallization can lead to compromise solutions, where a small decrease in crystal yield makes it possible to achieve a more environmentally desirable design. The formulation has also been applied to the crystallization of ibuprofen, under similar conditions, expressing the versatility of the general approach. Whereas it was found that integrating cooling and antisolvent crystallization can significantly improve the crystal yield of lovastatin, this hybrid approach and antisolvent crystallization is seen to offer similar performance in the case of ibuprofen. Furthermore, the solubilities of the two APIs, in the respective optimal solvent mixtures, have different responses to a change in temperature; for ibuprofen, the solubility curve shifts to improve the crystal yield and solvent consumption, whilst for lovastatin, the opposite is true. As such, the general methodology proposed in our current work is particularly powerful in highlighting optimal solutions across a range of API molecules and conditions, taking into account the subtle impacts of different chemistries.

The simultaneous design of the solvent mixture and operating temperature led to improvements compared to those achieved when the temperature range is fixed. Moreover, it was shown that maximizing the temperature range may not always result in the optimal operating conditions—the application of powerful but volatile solvents or antisolvents can instead improve the outcome of a crystallization, whilst reducing the initial operating temperature. In both case studies, a better performance is also found for the hybrid cooling and antisolvent crystallization techniques compared to standalone methods. Permitting the use of solvent mixtures throughout the entire crystallization introduces the possibility for the process to utilize all regions of the API solubility curve, potentially increasing crystal yield and reducing solvent consumption. Additionally, the proposed method is applicable to any number of mixture components and could thus be expanded to model ternary solvent mixtures, such as those where residual water may still be present following upstream solvent swaps or to model the impact of impurities, provided their molecular structure is known. Because the proposed approach is derived from the generic multicomponent framework of Jonuzaj *et al.*,^24^ it is in fact possible to optimize the identity of additional components. Finally, we find it remarkable that high performance (high yields and low solvent consumption) can be achieved using a relatively limited number of solvents. This may be due to some extent to the fact that the solvents considered cover several chemical classes. As more group-interaction parameters are developed for the SAFT-γ Mie equation of state,^30^ the range of solvents can be expanded further.

Because of the necessity to produce a flexible framework for solvent design in the pharmaceutical industry, other considerations are required to expand this type of study. Whilst the toxicity of solvents is used to shortlist candidates initially for the case studies explored here, it is important to consider other HSE aspects, such as the flammability or reactivity of solvents. This can be achieved by including additional GC methods in the formulation,^16^ selecting solvents based on the ICH classification^50^ or by applying safety indicators^11^ to the list of candidate solvents to screen them;^17^ Jonuzaj *et al.*(51) have recently investigated the effects of such a choice on crystallization metrics. Furthermore, although it is important to design efficient crystallization processes, problems can arise downstream from the morphology of the final crystal form, such as needle- or plate-like particles inhibiting filtration systems. This has been the subject of earlier work,^16^ which concentrated on the correlation between solubility parameters and crystal growth rates, but would benefit from a more mechanistic description of solvent effects on shape. Finally, it would be advantageous to take into the account wider process impacts of the use of solvents. This includes capital and operating costs—for instance, energy costs arising from solvent recovery and waste treatment—which may result in different cooling/antisolvent crystallization designs. It also includes broadening the scope of the processing steps considered to take into account isolation^51^ as well as synthesis, which has typically been considered separately from crystallisation.^52,53^

## Appendix

The SAFT-γ Mie group-contribution approach was used in this work to model the solubility of lovastatin. The like-group parameter values used in this model are shown in Table 8. The group-group dispersive interaction energies and repulsive exponents used in the model are given in Table 9. The group-group association energies and bonding volume parameter values used in the model are provided in Table 10.

**Table 8:** Like-Group Parameter Values for Use within the SAFT-γ Mie Group-Contribution Approach^a^

*k*	group *k*	*v*_*k*_	*S*_*k*_	λ_*kk*_^r^	λ_*kk*_^a^	σ_*kk*_/Å	(ϵ_*kk*_/*k*_B_)/K	*N*_st,*k*_	*n*_*k*,H_	*n*_*k*,e_1__	*n*_*k*,e_2__
1	CH_3_	1	0.57255	15.050	6.0000	4.0773	256.77				
2	CH_2_	1	0.22932	19.871	6.0000	4.8801	473.39				
3	CH	1	0.072100	8.0000	6.0000	5.2950	95.621				
4	CH_2_=	1	0.44890	20.271	6.0000	4.3175	300.90				
5	CH=	1	0.20037	15.974	6.0000	4.7488	952.54				
6	cCH_2_	1	0.24751	20.386	6.0000	4.7852	477.36				
7*	CH_2_OH	2	0.58538	22.699	6.0000	3.4054	407.22	2	1	2	0
8*	CHOH	1	0.37926	18.185	6.0000	4.5381	599.66	2	1	2	0
9	COO	1	0.65264	31.189	6.0000	3.9939	868.92	1	0	2	0
10	CH_3_COCH_3_	3	0.72135	17.433	6.0000	3.5981	286.02	3	1	1	1
11	H_2_O	1	1.0000	17.020	6.0000	3.0063	266.68	2	2	2	0

a *v*_*k*_, *S*_*k*_, and σ_*kk*_ are the number of segments in group *k*, the segment shape factor, and the segment diameter, respectively; λ_kk_^r^ and λ_*kk*_^a^ are the repulsive and attractive exponents, and ϵ_*kk*_ is the dispersive energy of the Mie potential characterizing the interaction of two *k* groups; *N*_st,*k*_ is the number of association site types on group *k*, and *n*_*k*,H_, *n*_*k*,e_1__, and *n*_*k*,e_2__ are the numbers of association sites of types H, e_1_, and e_2_, respectively. Rows marked with an asterisk contain parameter values introduced in our current work. The parameter values for groups without an asterisk are taken directly from Dufal *et al.*(54)

**Table 9:** Group–Group Dispersive Interaction Energies and Repulsive Exponents for Use within the SAFT-γ Mie Group-Contribution Approach^a^

*k*	*l*	group *k*	group *l*	(ϵ_*kl*_/*k*_B_)/K	λ_*kl*_^r^	*k*	*l*	group *k*	group *l*	(ϵ_*kl*_/*k*_B_)/K	λ_*kl*_^r^
1	1	CH_3_	CH_3_	256.77	15.050	4*	7	CH_2_=	CH_2_OH	375.51	CR
1	2	CH_3_	CH_2_	350.77	CR	4*	8	CH_2_=	CHOH	449.83	CR
1	3	CH_3_	CH	387.48	CR	4	9	CH_2_=	COO	CR	CR
1	4	CH_3_	CH_2_=	333.48	CR	4*	10	CH_2_=	CH_3_COCH_3_	288.20	CR
1	5	CH_3_	CH=	252.41	CR	4*	11	CH_2_=	H_2_O	387.25	94.463
1	6	CH_3_	cCH_2_	355.95	CR	5	5	CH=	CH =	952.54	15.974
1*	7	CH_3_	CH_2_OH	333.20	CR	5	6	CH=	cCH_2_	398.35	CR
1*	8	CH_3_	CHOH	479.38	CR	5*	7	CH=	CH_2_OH	414.91	CR
1	9	CH_3_	COO	402.75	CR	5*	8	CH=	CHOH	540.83	CR
1	10	CH_3_	CH_3_COCH_3_	233.48	14.499	5*	9	CH=	COO	818.79	CR
1	11	CH_3_	H_2_O	358.18	100.00	5*	10	CH=	CH_3_COCH_3_	437.75	CR
2	2	CH_2_	CH_2_	473.39	19.871	5*	11	CH=	H_2_O	332.21	17.309
2	3	CH_2_	CH	300.07	CR	6	6	cCH_2_	cCH_2_	477.36	20.386
2	4	CH_2_	CH_2_=	386.80	CR	6*	7	cCH_2_	CH_2_OH	CR	CR
2	5	CH_2_	CH=	459.40	CR	6*	8	cCH_2_	CHOH	554.50	CR
2	6	CH_2_	cCH_2_	469.67	CR	6	9	cCH_2_	COO	498.60	CR
2*	7	CH_2_	CH_2_OH	423.17	CR	6*	10	cCH_2_	CH_3_COCH_3_	352.19	CR
2*	8	CH_2_	CHOH	517.64	CR	6*	11	cCH_2_	H_2_O	347.48	28.497
2	9	CH_2_	COO	498.86	CR	7*	7	CH_2_OH	CH_2_OH	407.22	22.699
2	10	CH_2_	CH_3_COCH_3_	299.48	11.594	7*	8	CH_2_OH	CHOH	389.23	CR
2	11	CH_2_	H_2_O	423.63	100.00	7*	9	CH_2_OH	COO	CR	CR
3	3	CH	CH	95.621	8.0000	7*	10	CH_2_OH	CH_3_COCH_3_	338.47	CR
3	4	CH	CH_2_=	426.77	CR	7*	11	CH_2_OH	H_2_O	353.37	CR
3	5	CH	CH=	502.99	CR	8*	8	CHOH	CHOH	599.66	18.185
3	6	CH	cCH_2_	570.45	CR	8*	9	CHOH	COO	CR	CR
3*	7	CH	CH_2_OH	329.22	CR	8*	10	CHOH	CH_3_COCH_3_	340.81	CR
3*	8	CH	CHOH	0.00	CR	8*	11	CHOH	H_2_O	479.16	CR
3*	9	CH	COO	353.65	CR	9	9	COO	COO	868.92	31.189
3	10	CH	CH_3_COCH_3_	637.29	CR	9*	10	COO	CH_3_COCH_3_	547.44	CR
3	11	CH	H_2_O	275.75	CR	9*	11	COO	H_2_O	396.81	15.140
4	4	CH_2_=	CH_2_=	300.90	20.271	10	10	CH_3_COCH_3_	CH_3_COCH_3_	286.02	17.433
4	5	CH_2_=	CH=	275.75	CR	10	11	CH_3_COCH_3_	H_2_O	287.26	CR
4	6	CH_2_=	cCH_2_	CR	CR	11	11	H_2_O	H_2_O	266.68	17.020

a λ_*kl*_^r^ is the repulsive exponent and ϵ_*kl*_ is the dispersive energy of the Mie potential characterizing the interaction of two groups *k* and *l*. CR refers to parameter values that are determined using combining rules (see Dufal *et al.*(54)). Rows marked with an asterisk contain parameter values introduced in our current work, which will be the subject of a future paper. The parameter values for groups without an asterisk are taken directly from Dufal *et al.*(54)

**Table 10:** Group–Group Association Energies and Bonding Volume Parameter Values for Use within the SAFT-γ Mie Group-Contribution Approach^a^

*k*	*l*	group *k*	site *a* of group *k*	group *l*	site *b* of group *l*	(ϵ_*kl*,*ab*_^HB^/*k*_B_)/K	*K*_*kl*,*ab*_/Å^3^
7*	7	CH_2_OH	H	CH_2_OH	e_1_	2097.9	62.309
7*	8	CH_2_OH	H	CHOH	e_1_	2500.0	10.444
7*	8	CH_2_OH	e_1_	CHOH	H	1464.1	591.55
7*	10	CH_2_OH	H	CH_3_COCH_3_	e_1_	1844.8	991.95
7*	10	CH_2_OH	e_1_	CH_3_COCH_3_	H	686.93	585.99
7*	11	CH_2_OH	H	H_2_O	e_1_	621.28	425.00
7*	11	CH_2_OH	e_1_	H_2_O	H	2153.2	147.40
8*	8	CHOH	H	CHOH	e_1_	2480.6	8.4740
8*	10	CHOH	H	CH_3_COCH_3_	e_1_	1186.9	731.08
8*	10	CHOH	e_1_	CH_3_COCH_3_	H	1323.1	635.37
8*	11	CHOH	H	H_2_O	e_1_	2289.1	63.813
8*	11	CHOH	e_1_	H_2_O	H	2140.9	19.478
9	11	COO	e_1_	H_2_O	H	1245.81	454.98
10	10	CH_3_COCH_3_	H	CH_3_COCH_3_	e_1_	980.20	2865.2
10	11	CH_3_COCH_3_	H	H_2_O	e_1_	1386.8	188.83
10	11	CH_3_COCH_3_	e_1_	H_2_O	H	1588.7	772.77
10	11	CH_3_COCH_3_	e_2_	H_2_O	H	417.24	1304.3
11	11	H_2_O	H	H_2_O	e_1_	1985.4	101.69

a ϵ_*kl*,*ab*_^HB^ is the association energy of the Mie potential characterizing the interaction of site *a* of group *k* and site *b* of group *l*, and *K*_*kl*,*ab*_ is the bonding-volume parameter for such an interaction. Rows marked with an asterisk contain parameter values introduced in our current work, which will be the subject of a future paper. The parameter values for groups without an asterisk are taken directly from Dufal *et al.*(54)
